# From Identification to Intelligence: An Assessment of the Suitability of Forensic DNA Phenotyping Service Providers for Use in Australian Law Enforcement Casework

**DOI:** 10.3389/fgene.2020.568701

**Published:** 2021-01-12

**Authors:** Lauren Atwood, Jennifer Raymond, Alison Sears, Michael Bell, Runa Daniel

**Affiliations:** ^1^Science and Research Unit, Forensic Evidence and Technical Services Command, New South Wales Police Force, Sydney, NSW, Australia; ^2^Forensic Analytical and Science Service, New South Wales Health Pathology, Sydney, NSW, Australia; ^3^Office of the Chief Forensic Scientist, Victoria Police Forensic Services Department, Melbourne, VIC, Australia

**Keywords:** forensic DNA phenotyping, intelligence, casework, law enforcement, massively parallel sequencing

## Abstract

Forensic DNA Phenotyping (FDP) is an established but evolving field of DNA testing. It provides intelligence regarding the appearance (externally visible characteristics), biogeographical ancestry and age of an unknown donor and, although not necessarily a requirement for its casework application, has been previously used as a method of last resort in New South Wales (NSW) Police Force investigations. FDP can further assist law enforcement agencies by re-prioritising an existing pool of suspects or generating a new pool of suspects. In recent years, this capability has become ubiquitous with a wide range of service providers offering their expertise to law enforcement and the public. With the increase in the number of providers offering FDP and its potential to direct and target law enforcement resources, a thorough assessment of the applicability of these services was undertaken. Six service providers of FDP were assessed for suitability for NSW Police Force casework based on prediction accuracy, clarity of reporting, limitations of testing, cost and turnaround times. From these assessment criteria, a service provider for the prediction of biogeographical ancestry, hair and eye colour was deemed suitable for use in NSW Police Force casework. Importantly, the study highlighted the need for standardisation of terminology and reporting in this evolving field, and the requirement for interpretation by biologists with specialist expertise to translate the scientific data to intelligence for police investigators.

## Introduction

Since the application of DNA analysis in forensic casework in the late 1980s, considerable technological advancements have resulted in an expansion of forensic DNA analysis capabilities. Currently, in the majority of operational forensic laboratories, the use of DNA evidence is heavily focused on identification using STRs, limited by the reliance on comparison to other STR-generated profiles stored in a DNA database or to a reference sample from a known suspect. A notable difference with inferring biogeographical ancestry (BGA) and externally visible characteristics (EVCs) of an unknown individual—referred to as Forensic DNA Phenotyping (FDP) or DNA Intelligence—is the capacity to provide DNA information in an investigation to assist with individual identification by generating leads without reliance on the availability of a comparison sample. FDP enables investigators to generate or re-prioritise a suspect pool based on an unknown sample, thereby providing investigative leads that could assist with the identification of the DNA donor using STR profiling (or other) techniques. Such intelligence can be applied to cold cases, unidentified human remains cases and disaster victim identification; all scenarios where the success of STR identification can often have additional limitations due to degraded, or poor quality, biological evidence. This methodology has been applied successfully in casework for approximately 15 years with some of the earliest reported cases being the Louisiana Serial Killer case (2004) (Touchette, 2003) and the 11M Madrid bomb attack (Phillips et al., 2009).

Prediction accuracy is essential for confidence in result outcomes when applying FDP to casework. The use of relevant and informative DNA markers for the traits of interest is of paramount importance. Secondly, the composition of the reference set that is used to train the analysis algorithms must be appropriate and relevant for the predictive trait. The populations contained within these datasets are often unknown to the user or may vary considerably in their representative construct applicable to the trait being tested (Cheung et al., 2018). In addition, the accuracy of the prediction is dependent on the prediction algorithm used. Admixture is an additional challenge in the prediction of BGA, and ongoing research continues to address interpretation and reporting for operational application (Jin et al., 2018). The technical limitations of BGA and EVC prediction, including the availability of a quality sample, genetic admixture, and available reference datasets, have been discussed at various lengths (Kayser, 2015; Schneider et al., 2019).

A number of forensically relevant panels have been developed to provide accurate predictions of an unknown individual’s EVCs and BGA (Walsh et al., 2011b, 2013; Al-Asfi et al., 2017; Phillips et al., 2019). However, service providers differ in their testing approach and reference sets used, which may be reflected in the result outcome and, ultimately, the prediction accuracy. From an operational perspective, confidence in results and outcomes stems not only from a technically acceptable prediction, but a result that also clearly defines the reliability of the conclusion, whilst considering the above limitations of testing and reporting outcomes.

In addition to prediction accuracy, and contextualising testing limitations, an operational need is for a service provider to generate a report that is appropriate for direct release to a non-scientific/non-specialist audience (hereafter referred to as a lay audience). Reporting of STR profiles uses statistics to demonstrate the strength of a match whereas the use of statistical analysis for FDP reporting is to demonstrate the confidence in the prediction. Therefore, translation of scientific outcomes of FDP to lay audiences has been shown to be variable, particularly compared to STR profile reporting (Scudder et al., 2020). However, it has been proposed that ongoing education is beneficial for lay audiences to gain an understanding and awareness of the method and its application in casework (Daniel, 2016; Raymond et al., 2017). As FDP is a new and developing technology to be embedded in operational use within the New South Wales (NSW) Police Force, it is pertinent that considerable attention is focused on ensuring accurate comprehension by investigators.

The aim of this study (conducted in 2017/2018) was to compare results obtained from six providers of FDP services for BGA and EVCs (hair, eye, skin colour, and age) to determine suitability for operational application to NSW Police Force casework. Established service providers of FDP, with recognised expertise within the forensic community, were canvassed and invited to participate in the study. All service providers (six) that consented and were able to participate in the time frame requested were included. The service providers encompassed both commercial and non-commercial laboratories and groups to generate BGA and EVC data. Known volunteer donors with BGAs and EVCs representative of the diverse Australian population were selected for this study. The assessment criteria for determining suitability of a service provider for operational application must be strict and aligned with accreditation, legal, ethical and moral expectations of both the NSW Police Force and the community at large. This study used the following three categories for assessment:

1. Prediction accuracy
2. Clarity of reporting
3. Ability to generate results from all samples

For the purpose of this study, focus is placed on available service providers who could potentially contribute to operational environment needs, and assessment was based on the number of accurate predictions as compared to self-declared traits, with consideration given to the limitations described above. All service providers used in this study have been de-identified.

To assess clarity of reporting, service providers were asked to provide their report results as required for standard casework, if applicable. The service providers were based in several different countries and therefore report for consumers with varying legislative requirements. The reports were assessed for both accuracy of scientific content by a subject matter expert (SME) and for comprehension by a lay audience, such as law enforcement personnel (e.g., detectives and investigators), in the context of the adversarial legal system in place within NSW. The SME was a forensic biologist employed within an operational policing and forensic agency, with extensive academic and research expertise in DNA intelligence. Three factors were considered when reviewing reporting styles: consistent language, ease of interpretation and overall clarity.

Finally, cost and turnaround time are an important consideration in the operational application of any specialist service; therefore, these parameters were also considered. Whilst these points were not specifically requested of service providers, the service request was made for “…within usual work timeframes…” and quotes for service provision were provided based on the pre-determined sample numbers that would be submitted.

## Materials and Methods

### Donor Selection, Sampling, and Collection of Data

Ten known donors of varied BGA and EVCs relevant to the Australian population (Table 1) were sourced voluntarily from within the Forensic Evidence and Technical Services Command (FETSC) of the NSW Police Force. All donors provided informed consent and were de-identified, 1–10. The study was conducted in compliance with the *National Health and Medical Research Council (2015)*, consisting of a series of guidelines developed in accordance with the *National Health and Medical Research Council Act 1992* (NHMRC guidelines) (National Health and Medical Research Council, 2015).

**TABLE 1:** Donor’s self-declared biogeographical ancestry (BGA) and externally visible characteristics (EVCs).

**Donor**	**BGA**	**Eye colour**	**Hair colour**	**Skin colour**	**Age (years)**
Donor 1	Non-admixed South Asian	Brown	Black	Olive	49
Donor 2	Non-admixed Pacific Islands	Brown	Black	Olive	39
Donor 3	Admixed European/Aboriginal	Hazel	Dark brown	Medium	25
Donor 4	Non-admixed Middle Eastern	Brown	Dark brown	Medium	47
Donor 5	Non-admixed East Asian	Brown	Black	Medium	44
Donor 6	Non-admixed Middle Eastern	Brown	Dark brown	Olive	49
Donor 7	Non-admixed European	Blue	Blonde	Fair/Pale	27
Donor 8	Non-admixed South East Asian	Brown	Black	Olive	59
Donor 9	Non-admixed East Asian	Brown	Black	Medium	35
Donor 10	Non-admixed European	Hazel	Red	Fair/Pale	52

*The degree of admixture was determined using the BGA declared by the donor over three generations.*

Participants were asked to self-declare the following BGA and EVC information, which was confirmed by an independent evaluator at the time of collection to ensure consistency (Table 1). Photographs of the donors’ face, eyes and hair were also obtained (not shown).

– BGA: self-declared over three generations (self, parents, maternal and paternal grandparents, as per general biological pedigree definition). The degree of admixture was determined by a SME based on the donor’s self-declared BGA over the three generations;
– Eye colour: self-declared using categories blue, grey, green, hazel and brown;
– Skin colour: self-declared, based on an area of their body not exposed to light at age 20. Skin colour categories were fair/pale, medium, olive and dark.
– Hair colour: self-declared at age 0–4 years, 20 years old and current age. Natural hair colour categories were fair/blonde, light brown, light red/ginger, dark red/auburn, dark brown and black.
– Hair greying: self-declared percentage of grey currently, and approximate age at which greying occurred.

All six service providers used in this study were de-identified, denoted A–F. Samples of saliva and blood were collected as instructed by the service provider. Table 2 outlines the DNA sample type for each provider. DNA extracts were prepared from saliva stained Whatman FTA^TM^ MiniCard from each donor by the NSW Health Pathology Forensic and Analytical Science Services laboratory. DNA extraction was performed using PrepFiler^®^ Automated Forensic DNA Extraction Kit [Thermo Fisher Scientific (TFS)] and quantification using the Quantifiler^®^ Trio DNA Quantification Kit (TFS). A 5-mm hole punch of the saliva-stained Whatman FTA^TM^ MiniCard was sent to Providers A and D and DNA extract to Providers B and C for downstream analysis. The blood-stained Whatman FTA^TM^ MiniCard was provided to Provider A only. Neat saliva samples in proprietary collection tubes were sent to Providers E and F. Providers A, E and F did not disclose the minimum quantity of DNA required to perform testing.

**TABLE 2:** Sample types, assays, platforms and analysis methods for BGA and EVC prediction as disclosed by the six service providers.

**Provider**	**A**	**B**	**C**	**D**	**E**	**F**
Sample type	Whatman FTA^TM^ MiniCard—Blood Whatman FTA^TM^ MiniCard—Saliva (5-mm hole punch)	DNA extracts from saliva on Whatman FTA^TM^ MiniCard	DNA extracts from saliva on Whatman FTA^TM^ MiniCard	Whatman FTA^TM^ MiniCard—Saliva (5-mm hole punch)	Provider E^®^ and F^®^ DNA Collection Kit	

BGA	Marker panel and genotyping platform • Custom 41-plex SNP panel on MiSeq FGx (Illumina^®^) • mtDNA control region (Sanger) • PowerPlex^®^ Y23 (Promega) Analysis • SNIPPER, PCoA, STRUCTURE • EMPOP database • Y-HRD database	Marker panel and genotyping platform • Precision ID Ancestry Panel (TFS) (165 autosomal SNPs) • Ion PGM^TM^ System (TFS) • Ion Chef^TM^ (TFS) • Ion 316^TM^ v2 BC chips (TFS) • Ion PGM^TM^ Hi-Q^TM^ View Sequencing (TFS) Analysis • HID SNP Genotyper Plugin (TFS) • *STRUCTURE* • PCoA (Microsoft R)	Marker panel and genotyping platform • Custom marker panel (144 autosomal SNPs, panel in development) • AmpF*l*STR^TM^ Y Filer^TM^ PCR amplification kit (TFS) • Ion GeneStudio S5 System (TFS) Analysis • *Genotyper* software (TFS) • *Modified Genotyper* software (TFS) • NEVGEN Y-DNA Haplogroup Predictor • *Haplogrep* and EMPOP *Emma.* Phylotree v.17 • mtDNA analysis • SNIPPER, PCoA, STRUCTURE	Marker panel and genotyping platform • ForenSeq DNA Signature Prep Kit (Verogen) (231 STRs and SNPs) • MiSeq FGx (Verogen) Analysis • ForenSeq UAS (Verogen)	Undisclosed	

EVCs	Marker panel and genotyping platform • IrisPlex assay • SNaPshot^®^ Multiplex Kit (Applied Biosystems^TM^) • 3130xl Genetic Analyzer (Applied Biosystems^TM^) Analysis • IrisPlex Webtool (Erasmus)	Marker panel and genotyping platform • Ion Ampliseq^TM^ DNA Phenotyping Panel—24 SNPs (TFS) • Ion Chef^TM^ System (TFS) • Ion 314^TM^ v2 BC chips (TFS) • Ion PGM^TM^ Hi-Q^TM^ View Sequencing (TFS) Analysis • HIrisPlex Webtool (Erasmus)	Marker panel and genotyping platform • In-house multiplexes (incorporates HIIrisPlex SNPs) Analysis • SNIPPER • IrisPlex Webtool (Erasmus)	Marker panel and genotyping platform • ForenSeq DNA Signature Prep Kit (Verogen) (231 STRs and SNPs) • MiSeq FGx (Verogen) Analysis • ForenSeq UAS (Verogen)		

Age	Marker panel and genotyping platform • Custom marker panel (two multiplexes; 7plex and 5plex) • MiSeq FGx (Verogen) Analysis • Statistical models SVMp, LASSO, ANN					

Minimum Quantity	Undisclosed	1 ng total DNA	300 ng total DNA	4 ng total DNA (20 μl of 0.2 ng/μl)	Undisclosed	

### Analysis by Service Providers

The testing undertaken by each provider is shown in Table 2, in addition to the marker panels, genotyping platforms and analysis methods used as indicated in the provider’s results report or as declared by the provider. Providers B, C, and D all tested for eye colour, hair colour and BGA. Skin colour and age prediction were only tested by Providers C and A, respectively. Providers E and F only generated results for BGA and the marker panels, genotyping platforms and analysis methods used were not disclosed. The results from these providers were sent directly to the donors. The donors then chose to provide the results for this study. A SME and operational forensic scientists (biologists) assessed the results, analysis, prediction accuracy and reporting from the providers.

## Results

The summarised results generated by the service providers were assessed using three main criteria: prediction accuracy, clarity of reporting and ability to generate results from all samples. Additional criteria used to assess the providers included cost and turnaround time for analysis and reporting.

It was known prior to commencing this study that Providers E and F did not conduct analysis of EVCs and that their service does not include analysis of casework samples. However, these providers were included in the study for comparative purposes to assess variation in BGA prediction, accuracy and reporting styles between the service providers. A summary of the prediction performance for all service providers is shown in Supplementary Table 1.

### Prediction Accuracy

The prediction accuracy of each service provider for eye colour, hair colour, skin colour, age and BGA, respectively, is shown in Table 3. The prediction accuracy (%) indicates the number of correct predictions of the total predictions made. As indicated, results were not obtained, or not available, for all samples and testing type.

**TABLE 3:** The prediction accuracy (%) of each service provider for eye, hair and skin colour, BGA, and age.

	**Provider A**	**Provider B**	**Provider C**	**Provider D**	**Provider E**	**Provider F**
Eye colour	80%	90%	89%*	86%*	–	–
Hair colour	–	80%	80%	86%*	–	–
Skin colour	–	–	50%	–	–	–
BGA	90%	100%	60%	50%	100%	100%
Age	25%*	–	–	–	–	–
Cost/sample (USD)	$682	$556	$802	$164	$96	$96
Turnaround time (days)	66	30	114	21	28	28

– Service not provided. * Result not provided for all 10 donors. Costs as at 2017/2018 pricing.

#### Eye, Hair, and Skin Colour

Four of the six providers conducted eye colour analysis. Only two providers (Providers A and B) generated results for all 10 donors. Provider B achieved the highest predication accuracy for eye colour (90%) followed by Provider C (89%). However, the eye colour of donor 3 was not predicted correctly by any of the providers. Donor 3’s self-declared eye colour was hazel; however, it was predicted to be blue by all providers. Categorised as an intermediate eye colour, hazel eye colour has an expected prediction accuracy of 74% using Irisplex SNPs (Walsh et al., 2011a,b; Walsh et al., 2012, 2013). However, donor 10’s hazel eye colour was correctly predicted by Provider B but not by Provider A. Providers C and D did not return eye colour results for this sample. Self-declared brown and blue eye colours were correctly predicted.

Three of the six providers tested for hair colour. Only two providers (Providers B and C) generated results for all 10 donors. Provider D achieved the highest prediction accuracy for hair colour (86%). Providers B and C both achieved a hair colour prediction accuracy of 80%. The incorrect results generated by Provider B were for dark brown and blonde hair predicted to be black and brown, respectively. Provider C incorrectly predicted dark brown hair as red for donors 3 and black for donor 4. Provider D incorrectly predicted blonde hair as brown for donor 7.

Skin colour was only tested by Provider C and results were obtained for all 10 donors. However, the skin colour prediction accuracy was 50% with the incorrect predictions being for olive or medium skin colours predicted as white for donors 1, 3, 4, 6, and 8.

#### Age

**FIGURE 1:**
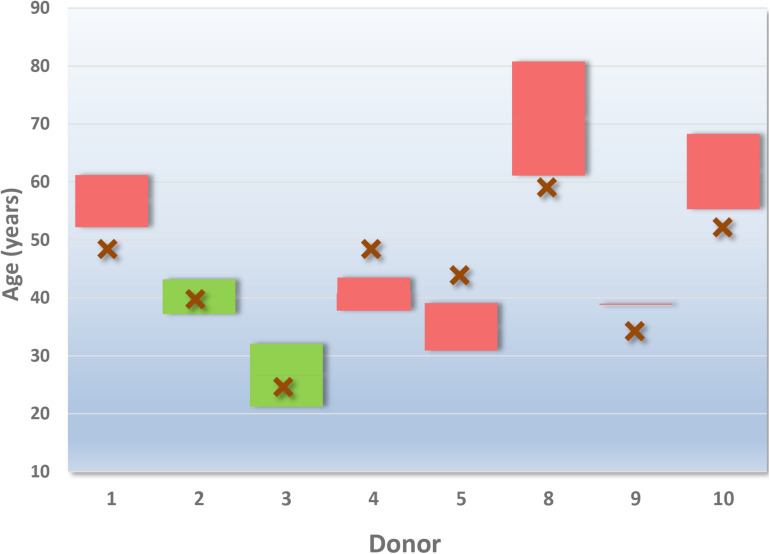
Provider A age predictions for 8 of the 10 donors. The declared age of the donor is indicated by an “X” (red). The boxes outline the predicted age range given by the service provider (reported by the provider as a “95% confidence interval”). Green boxes encompass a correct prediction. Red boxes indicate an incorrect prediction for the donor.

Figure 1 displays the age of the eight donors tested, against the predicted age range given by Provider A. Table 4 provides an example of provider A’s reporting style of predicted age ranges presented as a “mean age” and age range with a 95% confidence interval (as reported by the provider). The overall age prediction accuracy was 25%. Figure 1 demonstrates the variation in the age ranges provided across the donors. Although a small dataset, no trends in relation to correct predictions of older or younger donors were observed, nor were the predictions consistently below or above the correct age. A decrease in predicted age range did not correlate with an increase in successful age prediction.

**TABLE 4:** Provider A reporting style for eye colour, age, and BGA prediction.

**Provider A**	**Eye colour**	**Age**	**BGA**
Reported result	Predicted phenotype: Brown Prediction error: < 1%	Mean age: 56.7 95% Confidence Interval: 52.2–61.2	This sample is most likely from a South Asian population, such as Pakistan, but the origin could also be within an area that includes Pakistan and Iran, or less likely be within an area extending into Iraq or India. This prediction is not excluded by the Y-chromosome analysis and the STRUCTURE plot that shows an admixed population typical of some individuals in our reference pakistani population

#### BGA

All six providers tested for BGA. Providers A, B, C, D, and F returned results for the 10 donors. Providers B, E and F achieved the highest prediction accuracy for BGA (100%) followed by Provider A (90%). Results from Providers E and F were disseminated directly to the donors. Donor 2 did not return their BGA results from Provider E for inclusion in this study. Provider E did not generate a result for Donor 10.

The assays and genotyping platforms used by the providers varied greatly and included SNaPshot assays, massively parallel sequencing assays and high-density SNP arrays (Table 2). Therefore, the markers analysed, reference sets and prediction algorithms also varied, not allowing a direct comparison of prediction accuracies between providers. Additionally, the reporting styles of the providers ranged from referring only to geographic ancestry to including statements about ethnicity. However, of the traits predicted in this study, the highest prediction accuracies (100%) were generated for BGA prediction from three of the six providers. BGA prediction was offered by all service providers.

### Reporting Clarity

The service providers were instructed to provide a report that did not require additional interpretation and could be disseminated directly to investigators. Therefore, ease of comprehension by a lay person was a primary consideration in our assessment. Examples of reported results provided below have been selected to best represent the variation in reporting from service providers and to reflect the challenges associated with interpretation of these results. It was known prior to commencing this study that due to service capabilities, Provider D would only provide the genotyping platform’s onboard analysis software output without interpretation of the results. Comparative analysis of reporting was categorised into three key components: consistent language, ease of interpretation by a lay audience and overall clarity.

All service providers reported EVC results using consistent language within their reports. However, the reporting style for EVC results varied greatly between providers. The reported predicted phenotype is indicated as correct or incorrect based on comparison to the donor’s self-declared EVC.

To compare the donors’ self-declaration to the categories reported by service providers, the following process was applied when assessing the accuracy of hair and eye colour predictions:

(i) Self-declared brown (light or dark) hair colour: Any service provider predictions of “brown,” “dark brown” or “light brown” were recorded as correct.
(ii) Self-declared “light red or ginger” and “dark red or auburn” hair colour: Service provider predictions of “red” were recorded as correct.
(iii) Service provider predictions indicating a range of hair colours for a donor (e.g., Brown/Black) were recorded as correct if *any* of the hair colours predicted matched the donor’s self-declaration.
(iv) Self-declared “green,” “grey,” or “hazel” eye colours: Service provider predictions of “intermediate” eye colour were recorded as correct.

To compare the donor’s country specific self-declaration of BGA (e.g., Chinese, British, Turkish) the donor’s ancestry was reclassified to a sub-geographic region (e.g., East Asia, European, Middle East). In the case of the admixed donor (Table 1), the authors accepted a BGA prediction as correct based on the degree of admixture and the dominant ancestral geographic region declared, in deference to the lack of informative markers and individuals within the reference sets representative of the Australian Indigenous population (at the time of the study). The providers did not supply a list of countries specified within each sub-geographic region/population groups tested; therefore, the United Nations statistics division’s definition of sub-geographic regions was used (United Nations, 1999).

#### Provider A

The language used by Provider A to report eye colour and age for all donors was consistent throughout the report (Table 4). Provider A’s reporting style of eye colour was interpretable by a lay person. However, an explanation of the calculation for the prediction error rate was not provided. Provider A used the Erasmus IrisPlex/HIrisPlex predictor for eye colour phenotyping. At the time of reporting, the *IrisPlex & HIrisPlex DNA Phenotyping Webtool User Manual Version 1.0* (Forensic Molecular Biology Department of Erasmus MC, n.d.) states that this tool received “*overall prediction accuracies*” of 94% for blue eyes, 74% for intermediate eye colour and 95% for brown eye colour. Provider A commonly reported prediction errors of < 1%. Whilst this may be correct from the service provider’s perspective, without explanation of how this error rate was determined, there is potential for an investigator to incorrectly assume that the eye colour predictions from Provider A have a > 99% accuracy.

A discrepancy in eye colour prediction accuracy was observed between Providers A and B for donor 10. Although the two providers used the same eye colour markers and webtool, different genotyping methods were used [Provider A used an IrisPlex SNaPshot assay and manual interpretation whilst Provider B used a HIrisPlex-based MPS assay and automated interpretation (Table 2)]. These differences may explain Provider A’s incorrect prediction. However, as Provider A did not provide the genotype data, it was not possible to determine whether the incorrect prediction of Provider A was a result of a genotyping error or differences in interpretation thresholds and reporting criteria applied by each provider.

Although the reported result for age did not require further interpretation, the age ranges varied considerably from ∼5 months (Donor 9) to ∼19 years and 8 months (Donor 8). Without additional explanation, this reporting style may result in law enforcement personnel associating a degree of confidence with the relative size of the age range reported. For example, the larger the age range reported (i.e., 19 years and 8 months), the less confident an investigator might be in the prediction of age for that donor, and vice versa.

Provider A’s BGA results used varying language throughout their report. The use of terminology (i.e., “very confident”) was inconsistent within and between the donor results. Several of the summaries of donor results reported by Provider A were unclear and, at times, contradictory. For example, “*This sample is likely from an Asian population, but it is not typical of East Asian or South Asian populations. An East Asian and South-East Asian origin is suggested by mtDNA […] STRUCTURE reveals an admixture with a major East Asian and a minor South Asian contribution.*”

Provider A also used terminology in their report that infers race and skin colour rather than just geographic origin of the donor; e.g., “*this is a Caucasian individual with white European ancestry.*” This terminology was not used consistently in the report, as two additional donors with similar ancestry to donor 3 were not described in this manner. The term “Caucasian” is widely misunderstood, used as a synonym for “white” and holds no contemporary geographic links (Freedman, 1984; Bhopal and Donaldson, 1998). This combined with Provider A’s “*… white European*” classification highlights the need for standardised terminology when reporting BGA. It is also noted here that there is use of language that infers skin colour, when no such test had been performed.

#### Provider B

Provider B used consistent language throughout the reporting of all EVCs and BGA (Table 5). Their results were readily understandable by a lay audience. Provider B used terms common in verbal scales (“likely,” “very likely”) to report the EVC and BGA predictions. Provider B’s report did not indicate whether specific criteria were applied or predictive values were used to support this terminology. However, consistent use of these terms allowed comprehension of, and increased confidence in, the results by a lay audience.

**TABLE 5:** Provider B reporting style for eye colour, hair colour, and BGA prediction.

**Provider B**	**Eye colour**	**Hair colour**	**BGA**
Reported result	The donor of this DNA is most likely to have brown eyes	The donor of this DNA is most likely to have dark brown hair	The donor of this DNA has a majority ancestral genetic contribution from Europe. Examples include Hungary, Greece and Denmark. They are more likely to have European ancestry than any other continental BGA*. They are likely to have a majority of ancestors (e.g., parents, grandparents) from Europe.

*BGAs include African, Middle Eastern, European, South Asian, East Asian, Oceanian, indigenous American.

#### Provider C

Eye colour was determined via the output from two different tests Snipper eye and Erasmus eye (as reported by the provider), resulting in likelihood ratio (LR) and *p*-value statements with a predicted colour listed in bold text (Table 6). As Provider C did not combine the results into a single prediction, it was determined that the emphasised eye colour would be understood as the predicted phenotype from Provider C. In every case, the predictions from the *Snipper* eye and *Erasmus* eye tests concurred. It is not known how discordant results would be reported should they occur. Similarly, for hair and skin colour prediction, a LR was provided with a colour emphasised. Again, it was assumed that the emphasised colour was the prediction given by Provider C.

**TABLE 6:** Provider C reporting style for eye colour, hair colour, skin colour, and BGA prediction.

**Provider C**	**Eye colour**	**Hair colour**	**Skin colour**	**BGA**
Reported result	*Snipper* eye*:* 9,000 times more likely Brown than Intermediate *Erasmus* eye*: p*-value of 0.977 for Brown eye colour	*Snipper* hair: 1,130,436 times more likely Dark than Fair; insufficient predictive value for Brown vs. Black differentiation	*Snipper* skin: 649,715 times more likely White than Intermediate	Eastern european or Middle eastern ancestry

Provider C used consistent language throughout the reporting of all EVCs and BGA. However, it was observed that Provider C reported the same BGA result “*Eastern European or Middle Eastern ancestry*” for half of the donors in the study, with two correct predictions.

#### Provider D

As indicated previously, the reports from Provider D were solely based on instrumentation output of BGA and eye and hair colour analysis without further interpretation or reporting (Table 7). Therefore, the prediction results were determined by a SME and forensic biologists within the project team.

**TABLE 7:** Provider D reporting style for eye colour, hair colour, and BGA prediction.

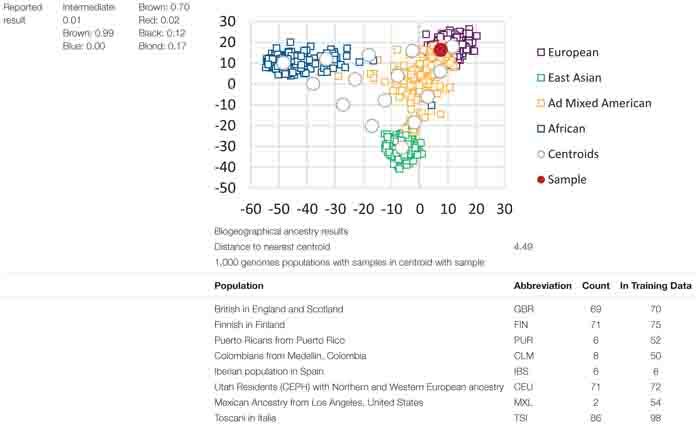

Provider D did not indicate a singular predicted eye or hair colour. A range of prediction values assigned to the categories of “Intermediate,” “Brown” and “Blue” for eye colour, and “Brown,” “Red,” “Black” and “Blond” for hair colour was provided. The investigator would be required to assume that the highest reported predictive value indicated would be the predicted phenotype.

As expected, the instrumentation output ensured that Provider D’s results were reported consistently, with the same language and presentation of result throughout the report. The BGA predictions were interpreted by the project team by selecting the population cluster where the donor sample was indicated on the graph. For example, in the case of the donor shown in Table 7, due to the overlapping population clusters, it was determined that the predicted BGA was European/Admixed American. The potential difficulties for law enforcement personnel to accurately grasp the instrumentation output highlight the need for expert interpretation and reporting.

#### Provider E

The results from Provider E were presented in a tabular style with percentages given for continental groups (e.g., European—94.5% and South Asian—1.4%). Sub-population groups were also listed under each continental group (e.g., British and Irish—84.5%, West African—1.2%). Due to copyright requirements from the provider, an example result is not shown here. Provider E used consistent language and reporting style across all 10 donors. The results reported were easily interpretable for a lay audience.

#### Provider F

Similar to Provider E, Provider F listed percentages against population groups but focused on the sub-populations rather than continental groups (e.g., Great Britain—34%, Europe West—17%, and so on). “Low confidence regions” were also listed. Additionally, Provider F supplied a global map with the “ethnicity estimate” (their terminology) presented as shaded circles over the relevant areas. Likewise, due to copyright requirements, the reporting style of Provider F is not shown here.

Provider F used consistent language and reporting style across all 10 donors. The results reported did not require additional interpretation. The use of a map as a visual aid as seen in Provider F’s reporting assisted in understanding the results. This reporting approach for law enforcement agencies may avoid misinterpretation of geographical regions and terminology. Any “low confidence regions” stated by Provider F were excluded from the assessment of Provider F’s predictive ability for BGA.

### Ability to Generate Results From All Samples, Costs, and Turnaround Time

Provider C returned eye colour prediction results for 90% of donors. Provider D produced eye and hair colour results for 70% of donors and Provider E was unable to return a BGA result for Donor 10. All other providers were able to return results for 100% of samples tested. All service providers were administered the quantity and quality of the DNA samples requested (Table 2). No explanation was provided by those service providers unable to return results for all samples.

Table 3 lists the costs and turnaround times for each provider. However, a direct comparison of the cost and turnaround time was not possible as the types of services, assays and technology used by each provider were often different.

## Discussion

The criteria applied to assess the six service providers in this study were selected to determine suitability for the application of FDP to casework samples. The service providers analysed samples from 10 donors (where possible) that self-declared their BGA, eye, hair and skin colour and age. This study indicated that the prediction accuracies and validated methodologies for BGA, hair and eye colour were appropriate for application to casework. Additional EVCs tested (skin colour and age) required more extensive research and development to increase the prediction accuracies. However, the authors note that considerable progress has been made on age and skin colour prediction since this study was conducted.

The donor samples received by the service providers were pristine, high source saliva or blood samples, unlike samples routinely encountered in casework. Casework samples often collected from trace evidence are of compromised quality (degraded). It is unlikely that casework samples will exceed the DNA quantity or quality of a sample retrieved directly from the donor source (e.g., a pristine or reference sample). Therefore, the inability to generate results from the donor samples was a point of consideration.

Of the four providers (A, B, C, and D) that could service law enforcement, based on the findings from the trial of 10 known donors using the criteria outlined in this study, Provider B was deemed suitable for use in NSW Police Force casework. A high prediction accuracy was observed for eye colour (90%), hair colour (80%), and BGA (100%). Provider B’s reporting style also satisfied the clarity assessment with clear, concise and effectively communicated results. Generating results occurred within a suitable time frame (30 days) and average cost/sample. Results were provided for all donor samples.

As a result of this study, FDP was incorporated into routine NSW Police casework. Future considerations for full operationalisation include assimilation into a quality framework with regular proficiency testing as per routine forensic analyses and accreditation requirements. Utilising the lessons learnt, the SME was engaged to interpret Service Provider B’s data analysis output and report the results using a reporting style template developed for dissemination directly to investigators.

Based on interaction and feedback from investigators, the ideal reporting template would include clear and concise language comprehensible by a non-expert/lay audience. The performance of the assays, etc. would be assessed by the scientific expert; therefore, the test characteristics mentioned previously do not require interpretation by the lay audience. Although indicating the accuracy of the prediction should be communicated to the investigator, language used to communicate this would not use scientific or specialist terms such as LR. Exclusions may be reported where possible and the limitations of the tests should be clearly indicated. FDP reporting style and dissemination of the results are important considerations for law enforcement agencies that will be addressed in a subsequent manuscript.

Observations made throughout this study have highlighted the need for caution and further discussion surrounding FDP, specifically the interpretation and reporting of results for law enforcement consumption. In general, greater contextual understanding of outcomes could be achieved through standardised reporting terminology. The key observed points from this study relate to (i) definition of sub-geographic regions for BGA predictions, (ii) avoidance of association of BGA prediction with an individual’s physical appearance, and (iii) standardisation of nomenclature for broader comprehension of results.

Regarding the issue of definition of sub-geographic regions, the reported BGA results are provided on a continent or sub-continent level. Most individuals are expected to refer their ancestry as a country-specific declaration (i.e., Chinese) as opposed to a continental or continental sub-regional scale (i.e., East Asian). This presents a challenge regarding how best to correlate the two in order to (1) define which countries lay within the reported sub-geographic region and (2) reach a consensus between the different service providers of how this should be defined and reported to achieve consistency.

A lay person’s interpretation of countries that may be included in a sub-geographic region, such as “East Asia” and “Middle East,” may be influenced by a number of factors such as the individual’s conscious and unconscious biases (life experience, education, social, and political context) (Samuel and Prainsack, 2018). To exacerbate the issue, definitions of countries included in sub-geographic regions are subject to change with shifting political and social influences/circumstances. It is recommended that each service provider provides a comprehensive list of countries within a region that align with their reporting of BGA prediction, or a map that would include their definitions of sub-geographic regions relevant to their reference populations.

EVC prediction accuracy assessment required alignment of the self-declared and reported eye and hair colour categories. In such a comparison, the differences in categories used are a potential source of error and highlight the need for standardised collection and reporting of EVCs to remove subjectivity. Future assessments may benefit from provision of defined self-declaration and reporting categories to both participants and providers to increase consistency.

A separate issue relates to the association of BGA assessment with an individual’s physical appearance. Prediction of the BGA of a donor is not the prediction of race, ethnicity or cultural background. It provides a prediction of the ancestral geographic or sub-geographic region of that donor. It is important to convey to law enforcement that although the affiliation between BGA prediction and assumption of physical appearance may align in some cases, BGA prediction does not imply the physical appearance of the DNA donor (Kayser and Schneider, 2012; Samuel and Prainsack, 2018).

The requirement for standardisation of nomenclature was the third issue highlighted from this study. Inferences of an individual’s BGA or EVCs can be made using FDP. However, this may be a probabilistic prediction depending on the trait of interest. Therefore, it is possible for the nature of the information to be misunderstood (Enserink, 2011; Cino, 2016; Samuel and Prainsack, 2018). The reporting of EVC results from providers in this study highlighted the need for standardised language to indicate the test performance (positive predictive value, negative predictive value, sensitivity, specificity) to assist with scientific interpretation. In addition, the service providers should provide the genotype data generated for every test undertaken to allow for independent verification of the results by the SME. Lay interpretation could be assisted by the provider indicating the % correct predictions per phenotype as a means of indicating potential error. Regardless of accurate predictions of BGA and EVCs by the service providers, equally as important is the delivery of the information.

The issues identified in this study support the involvement of a SME as a critical aspect of the interpretation and dissemination of FDP results to investigators. It was evident that providing an external report directly to investigators without SME review of the service provider’s analysis and interpretation increases the potential for misinterpretation. Given that this is a form of intelligence used to generate investigative leads, there is also potential for inadequate or expert review of the results to misdirect an investigation.

It is clear from these findings that there is merit in developing standardised nomenclature and reporting of DNA intelligence. Benefits of this approach would ensure that DNA intelligence can be more extensively integrated within law enforcement investigations, effectively communicated to investigators and to minimise the potential for misinterpretation.

## Data Availability Statement

The datasets presented in this article are not readily available because approval for access to datasets must be granted. Requests to access the datasets should be directed to LA, atwo1lau@police.nsw.gov.au.

## Ethics Statement

Ethical review and approval was not required for the study on human participants in accordance with the local legislation and institutional requirements. The patients/participants provided their written informed consent to participate in this study. Written informed consent was obtained from the individual(s) for the publication of any potentially identifiable images or data included in this article.

## Author Contributions

LA: data collation and analysis, logistics, background research, and writing—original draft. JR: conceptualisation, proposal, methodology, and writing—reviewing and editing. AS: methodology, logistics, and writing—reviewing and editing. MB: conceptualisation and supervision. RD: data interpretation and reporting, expert knowledge, methodology, and writing—reviewing and editing. All authors contributed to the article and approved the submitted version.

## Conflict of Interest

The authors declare that the research was conducted in the absence of any commercial or financial relationships that could be construed as a potential conflict of interest.
