# Supplementary material for: From Identification to Intelligence: An Assessment of the Suitability of Forensic DNA Phenotyping Service Providers for Use in Australian Law Enforcement Casework
Source: Front Genet. 2021 Jan 12;11:568701. doi: 10.3389/fgene.2020.568701 (PMC7835938; doi:10.3389/fgene.2020.568701)
Supplement: Supplementary file 1 [file Table_1.DOCX]

Supplementary Table 1 – A summary of prediction performance for all service providers

NA Result not reported from the service provider

* Sample not reported from the service provider

# Result not available

- Service not available


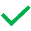
 Result correctly predicted


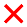
 Result incorrectly predicted

| **Eye Colour Prediction** | | | | | | |
| --- | --- | --- | --- | --- | --- | --- |
|  | ‘Provider A’ | ‘Provider B’ | ‘Provider C’ | ‘Provider D’ | ‘Provider E’ | ‘Provider F’ |
| Donor 1 | 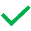 | 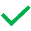 | 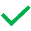 | 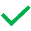 |  |  |
| Donor 2 | 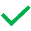 | 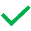 | 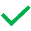 | NA |  |  |
| Donor 3 | 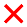 | 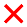 | 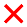 | 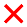 |  |  |
| Donor 4 | 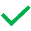 | 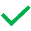 | 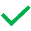 | NA |  |  |
| Donor 5 | 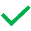 | 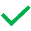 | 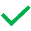 | 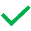 |  |  |
| Donor 6 | 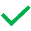 | 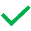 | 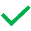 | 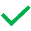 |  |  |
| Donor 7 | 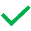 | 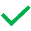 | 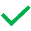 | 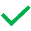 |  |  |
| Donor 8 | 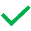 | 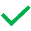 | 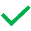 | 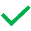 |  |  |
| Donor 9 | 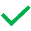 | 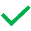 | 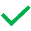 | 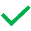 |  |  |
| Donor 10 | 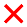 | 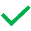 | NA | NA |  |  |
| Accuracy | 80% | 90% | 88.89% | 85.71% |  |  |
| **Hair Colour Prediction** | | | | | | |
|  | ‘Provider A’ | ‘Provider B’ | ‘Provider C’ | ‘Provider D’ | ‘Provider E’ | ‘Provider F’ |
| Donor 1 |  | 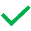 | 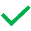 | 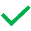 |  |  |
| Donor 2 |  | 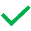 | 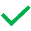 | NA |  |  |
| Donor 3 |  | 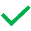 | 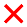 | 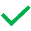 |  |  |
| Donor 4 |  | 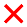 | 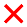 | NA |  |  |
| Donor 5 |  | 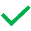 | 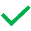 | 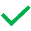 |  |  |
| Donor 6 |  | 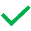 | 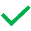 | 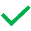 |  |  |
| Donor 7 |  | 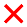 | 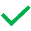 | 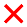 |  |  |
| Donor 8 |  | 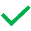 | 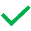 | 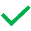 |  |  |
| Donor 9 |  | 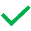 | 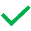 | 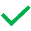 |  |  |
| Donor 10 |  | 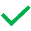 | 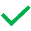 | NA |  |  |
| Accuracy |  | 80% | 80% | 85.71% |  |  |
| **Skin Prediction** | | | | | | |
|  | ‘Provider A’ | ‘Provider B’ | ‘Provider C’ | ‘Provider D’ | ‘Provider E’ | ‘Provider F’ |
| Donor 1 |  |  | 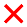 |  |  |  |
| Donor 2 |  |  | 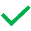 |  |  |  |
| Donor 3 |  |  | 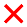 |  |  |  |
| Donor 4 |  |  | 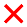 |  |  |  |
| Donor 5 |  |  | 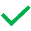 |  |  |  |
| Donor 6 |  |  | 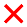 |  |  |  |
| Donor 7 |  |  | 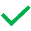 |  |  |  |
| Donor 8 |  |  | 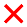 |  |  |  |
| Donor 9 |  |  | 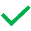 |  |  |  |
| Donor 10 |  |  | 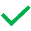 |  |  |  |
| Accuracy |  |  | 50% |  |  |  |
| **Age Prediction** | | | | | | |
|  | ‘Provider A’ | ‘Provider B’ | ‘Provider C’ | ‘Provider D’ | ‘Provider E’ | ‘Provider F’ |
| Donor 1 | 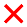 |  |  |  |  |  |
| Donor 2 | 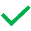 |  |  |  |  |  |
| Donor 3 | 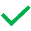 |  |  |  |  |  |
| Donor 4 | 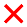 |  |  |  |  |  |
| Donor 5 | 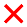 |  |  |  |  |  |
| Donor 6 | * |  |  |  |  |  |
| Donor 7 | * |  |  |  |  |  |
| Donor 8 | 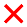 |  |  |  |  |  |
| Donor 9 | 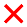 |  |  |  |  |  |
| Donor 10 | 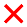 |  |  |  |  |  |
| Accuracy | 25% |  |  |  |  |  |
| **BGA Prediction** | | | | | | |
|  | ‘Provider A’ | ‘Provider B’ | ‘Provider C’ | ‘Provider D’ | ‘Provider E’ | ‘Provider F’ |
| Donor 1 | 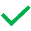 | 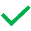 | 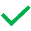 | 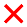 | 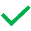 | 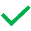 |
| Donor 2 | 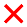 | 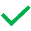 | 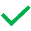 | 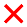 | # | 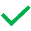 |
| Donor 3 | 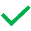 | 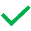 | 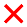 | 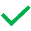 | 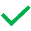 | 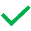 |
| Donor 4 |  |  |  |  |  |  |
| Donor 5 |  |  |  |  |  |  |
| Donor 6 |  |  |  |  |  |  |
| Donor 7 |  |  |  |  |  |  |
| Donor 8 |  |  |  |  |  |  |
| Donor 9 |  |  |  |  |  |  |
| Donor 10 |  |  |  |  | NA |  |
| Accuracy | 90% | 100% | 60% | 50% | 100% | 100% |
